# A two-step method for variable selection in the analysis of a case-cohort study

**DOI:** 10.1093/ije/dyx224

**Published:** 2017-11-10

**Authors:** P J Newcombe, S Connolly, S Seaman, S Richardson, S J Sharp

**Affiliations:** 1 MRC Biostatistics Unit, Cambridge, UK; 2 MRC Epidemiology Unit, Cambridge, UK

**Keywords:** Case-cohort study, survival analysis, variable selection, Bayesian variable selection, type 2 diabetes, fatty acids

## Abstract

**Background:**

Accurate detection and estimation of true exposure-outcome associations is important in aetiological analysis; when there are multiple potential exposure variables of interest, methods for detecting the subset of variables most likely to have true associations with the outcome of interest are required. Case-cohort studies often collect data on a large number of variables which have not been measured in the entire cohort (e.g. panels of biomarkers). There is a lack of guidance on methods for variable selection in case-cohort studies.

**Methods:**

We describe and explore the application of three variable selection methods to data from a case-cohort study. These are: (i) selecting variables based on their level of significance in univariable (i.e. one-at-a-time) Prentice-weighted Cox regression models; (ii) stepwise selection applied to Prentice-weighted Cox regression; and (iii) a two-step method which applies a Bayesian variable selection algorithm to obtain posterior probabilities of selection for each variable using multivariable logistic regression followed by effect estimation using Prentice-weighted Cox regression.

**Results:**

Across nine different simulation scenarios, the two-step method demonstrated higher sensitivity and lower false discovery rate than the one-at-a-time and stepwise methods. In an application of the methods to data from the EPIC-InterAct case-cohort study, the two-step method identified an additional two fatty acids as being associated with incident type 2 diabetes, compared with the one-at-a-time and stepwise methods.

**Conclusions:**

The two-step method enables more powerful and accurate detection of exposure-outcome associations in case-cohort studies. An R package is available to enable researchers to apply this method.

**Key Messages**

- The case-cohort study, where all cases are combined with a random subcohort, is a useful design when it is impractical to obtain measures of all variables in the entire cohort.
- There is a lack of guidance on variable selection methodology for case-cohort studies, i.e. how to detect which exposure variables are most likely to be truly associated with the outcome.
- We compare three possible strategies for variable selection, including the use of a recently published Bayesian variable selection (BVS) algorithm, which we have implemented in an R package.
- In simulation studies a two-step method, using BVS in the first step, demonstrated higher sensitivity and lower false discovery rates than other (one-at-a-time and stepwise) methods.
- The methods are exemplified using real data from the EPIC-InterAct case-cohort study.

## Introduction

Use of the case-cohort study design has become increasingly widespread in epidemiology in recent years^1^, often motivated by the impracticality of obtaining measures of all variables of interest on an entire cohort (e.g. if the variables include a panel of biomarkers which are expensive to measure). A case-cohort study for a particular outcome (e.g. disease or event) consists of a random subcohort selected from a cohort irrespective of outcome status, using a pre-defined sampling fraction, together with all incident cases of the outcome. Since all cases are included, the study has similar efficiency to a nested case-control study,^2^ but with the advantage that the same random subcohort can be used in future case-cohort studies for different outcomes.

Methods for estimating exposure-outcome associations in case-cohort studies, using an adaptation of standard Cox regression based on a weighted `pseudo-likelihood’ function, are well documented^3–5^ and commonly used.^1^ However, despite the fact that case-cohort studies often collect data on a large number of variables, there is a lack of guidance on methodology for variable selection, i.e. how to identify the subset of variables most likely to be causally associated with the outcome of interest. Accurate detection and estimation of causal associations is important in aetiological analysis and when there are multiple variables of interest, to inform future replication studies. In this work we describe and explore the application of three variable selection methods to data from a case-cohort study.

The first, simplest method is to analyse each variable one-at-a-time using Prentice-weighted Cox regression, and assess significance using a pre-determined multiplicity-adjusted *P*-value threshold. However, this ignores correlations among variables. Consequently, the number of causal associations, and the most suitable candidates for follow-up, is often unclear. For example, if a single causal variable is strongly correlated with many other variables, as is often the case in genomic data, all may result in similarly significant *P*-values due to confounding. Stepwise selection, the second method we consider, can be used to account for these correlations. However, stepwise selection procedures are well known to be both conservative and unstable, requiring arbitrary *P-*value thresholds for selection, and often leading to potentially spurious selections.^6–8^ Therefore the third method we investigate uses Bayesian Variable Selection (BVS), a method based on Bayesian sparse logistic regression which has previously been developed for cohort studies.^9^ Sparse (or penalised) regression^8^ is an area under active development in both the frequentist^8^^,^^10–12^ and Bayesian^9^^,^^13–15^ frameworks, which has been shown to result in more robust and accurate selections than stepwise methods.^7^

In the next section, we describe these three methods in more detail. Then in the section on comparison of methods, we use simulations to compare the performance of these methods, followed by a section applying all three methods to data from the EPIC-InterAct case-cohort study.

## Three methods for variable selection in the analysis of case-cohort studies

In the following descriptions, for an individual *i*, we use $x_{i}$ to denote measured values of each potential exposure variable of interest, and $d_{i}$ as a binary indicator that individual *i* experienced the event of interest (i.e. was a case) during the follow-up period.

### One-at-a-time method

Standard Cox regression defines a model for the hazard function of individual i as follows:

$$h_{i}\left(t\right)\;=\;h_{0}\left(t\right)e^{\beta_{\mathrm{HR}}x_{i}}\;$$

where $h_{0}\left(t\right)$ represents the baseline hazard function and $\beta_{\mathrm{HR}}$ a vector of log hazard ratios for each variable. Estimates of $\beta_{\mathrm{HR}}$ are obtained by maximizing the partial likelihood function:

$$L_{Cox}\left(\beta_{\mathrm{HR}}\right)\;=\;\underset{i}{\prod }\frac{e^{\beta_{\mathrm{HR}}x_{i}d_{i}}}{\sum_{j\in R_{i}}e^{\beta_{\mathrm{HR}}x_{j}}}$$

$R_{i}$ is the `risk set’ for individual *i*, i.e. the set of individuals still event-free and uncensored immediately before individual i’s event occurs.

Due to the over-representation of cases in a case-cohort study, various weighting schemes for the likelihood function have been proposed. Weights originally proposed by Prentice^3^ have been demonstrated in a range of scenarios to provide good approximations to parameter estimates that would have been obtained if data had been available for all individuals in the original cohort on which the case-cohort study was based.^16^

Using Prentice weights, cases outside the subcohort are not considered to be at risk until just before their event, and so are not included in the risk sets of earlier cases. Consequently, they only contribute to the denominator at the time of their event; the resulting `pseudo-likelihood’ function is:

$$L_{Prentice}\left(\beta \right)\;=\;\underset{i}{\prod }\frac{e^{\beta_{\mathrm{HR}}x_{i}d_{i}}}{e^{\beta_{\mathrm{HR}}x_{i}}+\sum_{j\in R_{i},j\neq i}e^{\beta_{\mathrm{HR}}x_{j}}}$$

Several robust variance estimators that account for the weighting in the pseudo-likelihood have been proposed.^3^^,^^5^^,^^17^^,^^18^ In this paper we use the method proposed by Prentice,^3^ which is the default in the `cch’ package in R.

We define the one-at-a-time method as fitting a separate Prentice-weighted Cox regression model to each variable in turn, and selecting all variables that are significant according to some pre-defined multiplicity-adjusted *P-*value threshold.

### Stepwise selection method

Stepwise selection aims to account for correlations between variables by using multivariable models to identify combinations of variables which are significantly associated with the outcome. We explore the use of a forward stepwise selection for Prentice-weighted Cox regression. The procedure is as follows.

- Starting with no variables in the model, we fit a separate univariable Prentice-weighted Cox model including each variable one by one. The variable (assuming there is one) with the most statistically significant *P*-value below a pre-defined inclusion threshold is selected.
- We fit separate multivariable Prentice-weighted Cox models including the variable selected at stage 1 and adding each remaining variable one by one. Of the remaining variables, the variable (assuming there is one) with the most statistically significant *P*-value below the inclusion threshold is selected.
- If a variable was added to the model at step (ii), all previously selected variables are checked to see if they still reach the inclusion threshold, and are dropped one by one if not, starting with the least significant.
- Steps (ii) and (iii) are repeated until none of the remaining variables have a *P*-value below the inclusion threshold when added to the model including the previously selected variables.

A common criticism of the stepwise variable selection is the arbitrariness in selecting a *P*-value threshold for inclusion. Here we will adopt the widely used threshold of 0.05 as default, but also consider results under a more liberal threshold of 0.1.

### Two-step Bayesian Variable Selection method

#### Step 1. Sparse BVS using multivariable logistic regression

Recall that $x_{i}$ denotes the measurements of all available variables for individual *i.* Under a multivariable logistic regression, the probability $\pi_{i}$ that individual *i*’s event is observed (i.e. $d_{i}\;=\;1)\;$is modelled as a log odds:

$$logit\left(\pi_{i}\right)\;=\;log\left(\frac{\pi_{i}}{1-\pi_{i}}\right)\;=\;\alpha +\beta_{OR}x_{i}$$

where $\beta_{OR}$ is a vector containing log-odds ratios for each of the measured variables. Logistic regression provides valid odds ratios for both prospectively collected data (e.g. a cohort study with a population-representative number of incident cases) and retrospectively collected data (e.g. a case-control study with equal numbers of cases and controls). Therefore logistic regression is applicable to case-cohort data, without the need for weighting to reflect over-representation of cases relative to the general population.

The multivariable logistic regression defined above includes all available variables. If there is a large number of variables, then the limited amount of information in the data will be spread too thinly and the significance of odds ratios will be unreliable and, in statistical terms, `over-fitted’ to the dataset at hand. Using a BVS algorithm, we model the probability that each variable v is selected, i.e. the corresponding log-odds ratio, $\beta_{OR,v}$, and x values are included in the multivariable regression. A sparse prior distribution is assumed for each variable’s selection probability, reflecting the belief that most variables will be irrelevant. This leads to the exclusion of many variables and thus reduces the problem of over-fitting, leading to more reliable inference on the relative significance of associations. BVS allows the calculation of posterior probabilities that each variable is selected, as well as probabilities for combinations of variables. A variety of formulations have been proposed;^19–21^ here we use Reversible Jump Markov Chain Monte Carlo (RJMCMC).^22^ Specifically, we assign a Beta prior distribution to the proportion of variables selected, θ:

θ∼Beta(1,P)

where P is the total number of variables. The marginal prior odds of any single variable being selected is 1/P, and therefore decreases with the total number of variables explored, providing an intrinsic correction for multiplicity.^23^ Further technical details on the Reversible Jump variable search algorithm we used are given in Newcombe *et al.*^9^

In addition to posterior probabilities of association, a `Bayes Factor’ for each variable can be calculated as the posterior odds of selection divided by the prior odds of selection. Intuitively, higher values of this ratio imply greater evidence of an association. Thresholds of 3–5 have been recommended for assessment of statistical significance.^24^

Note that we use the logistic model for Bayesian variable selection since we are not aware of any methodology that allows formal Bayesian inference to be drawn under a ‘pseudo’ likelihood with weights to reflect the over-representation of cases in case-cohort data. As explained above, the logistic model is valid in case-cohort data without the need for weighting. We chose not to use a standard Bayesian survival model, i.e. without weighting, since the resulting bias in hazard ratios from ignoring the case-cohort design would lead to inaccurate variable selections. Instead we estimate hazard ratios using a (non-Bayesian) weighted Cox regression in a second step, described below.

#### Step 2. Estimation of hazard ratios using multivariable Prentice-weighted Cox regression

Having identified a set of significant variables in Step 1, for example using a Bayes Factor threshold of 5, hazard ratios for each of these variables (adjusted for the others) can then be estimated by including them in a multivariable Prentice-weighted Cox model. Logistic regression assumes the censoring times are independent of the failure time and covariates x, whereas (Prentice-weighted) Cox regression requires the weaker assumption of independence only with the failure times. Therefore, we recommend leveraging the event times and censoring data, which are ignored by logistic regression, in the final estimation of effects.

## Comparison of methods using simulation

### Data-generating mechanisms

To compare the three methods described in above, we generated datasets for various scenarios, based on different numbers of variables and different sizes of correlations between variables. The assumptions for each scenario are summarised in Table 1.

**Table 1.** Assumptions used to generate artificial datasets used in the comparison of methods

Scenario	Number of variables	Pairwise correlations	Number of signals	HR signals	Baseline hazard function
					
1	20	0.2			
2	20	0.5			
3	20	0.8			
4	100	0.2			
5	100	0.5	5	1.1, 0.78, 1.48, 0.58, 2	Weibull(30,4)
6	100	0.8			
7	1000	0.2			
8	1000	0.5			
9	1000	0.8			

We considered M = 20, 100 or 1000 variables. For each member of a cohort of 20 000 individuals, we generated the values of the M variables from a multivariate normal distribution with zero means, unit variances and, in separate scenarios, correlation 0.2 (weak correlation), 0.5 (moderate correlation) or 0.8 (strong correlation) between each pair of variables. Each combination of M and strength of pairwise correlation led to nine simulation scenarios in total.

In every scenario, five variables were assumed to be truly associated with the outcome, and assigned log hazard ratios (log HRs) ranging in magnitude from log(1.1) to log(2) in equal steps (on a log scale), but with alternating directions, so there were three positive and two negative associations. Survival times were then generated based on these HRs and a baseline Weibull(30,4) hazard function, as used in Jones *et al.*^25^ Survival times were right-censored at a fixed time *C*, chosen so that 5% of events occurred before *C* for each scenario. Random censoring times were generated from an exponential distribution with rate –log(0.9)/C such that ∼10% of individuals were censored before *C.*

For each scenario we simulated data for 200 cohort studies, from which case-cohort datasets were generated, using a subcohort sampling fraction of 5%. The case-cohort datasets included on average 918 cases and 954 non-cases.

### Sensitivity and false discovery rate

We compared the three variable selection methods in terms of sensitivity, i.e. the proportion of `signal’ variables which were detected, and false discovery rate, i.e. the proportion of selected variables which were in fact `noise’ variables. For each scenario and method, the sensitivity over 200 simulations is shown in Table 2. For a fair comparison, the *P-*value threshold for the one-at-a-time method and the posterior probability threshold for the BVS method were chosen so that the resulting false discovery rates were the same as that of the stepwise method. Therefore, we compare sensitivity across the methods at a fixed false discovery rate.

**Table 2.** Sensitivity of variable selection methods, for each scenario

Method	Pairwise correlation between all variables		
	0.2	0.5	0.8
	**5 signals among 20 variables**		
One-at-a-time*	0.76 (0.01)	0.48 (0.01)	0.44 (0.01)
Stepwise	0.76 (0.01)	0.72 (0.01)	0.59 (0.01)
Two-step BVS*	0.89 (0.01)	0.84 (0.01)	0.73 (0.01)
	**5 signals among 100 variables**		
One-at-a-time*	0.69 (0.01)	0.44 (0.01)	0.40 (0.01)
Stepwise	0.76 (0.01)	0.71 (0.01)	0.59 (0.01)
Two-step BVS*	0.84 (0.01)	0.78 (0.01)	0.66 (0.01)
	**5 signals among 1000 variables**		
One-at-a-time*	0.64 (0.01)	0.41 (0.00)	0.34 (0.01)
Stepwise	0.77 (0.01)	0.70 (0.01)	0.56 (0.01)
Two-step BVS*	0.79 (0.01)	0.73 (0.01)	0.58 (0.01)

Mean sensitivity, the proportion of true signals selected, is displayed for 200 simulations with the corresponding Monte Carlo errors in brackets.

*Selection thresholds chosen to match the false discovery rate of the stepwise method in each simulation, for which a nominal *P*-value inclusion threshold of 0.05 was used.

The one-at-a-time method resulted in consistently lower sensitivity than the multivariable stepwise approach, in all scenarios. This clearly demonstrates a loss of efficiency from ignoring correlations between variables. The BVS method consistently resulted in the highest sensitivity, particularly for the scenarios involving 100 and 20 covariates where absolute sensitivity was increased by more than 5% and 10% relative to the stepwise method. Any loss of information due to ignoring times to event in the logistic BVS approach is apparently outweighed by use of a superior multivariable search strategy.

We also compared the false discovery rates of the three methods (Table 3) when the *P*-value and posterior probability thresholds for the one-at-a-time and BVS methods were used that resulted in the same sensitivity as the stepwise method. False discovery rates were similar for the BVS and stepwise methods in the 20 variable scenarios. However, the BVS selections consistently resulted in lower false discovery rates (often markedly so) for the harder 100 and 1000 variable scenarios. As expected, the one-at-a-time method had a higher false discovery rate than both multivariable methods.

**Table 3.** False discovery rates of variable selection methods, for each scenario

Method	Pairwise correlation between all variables		
	0.2	0.5	0.8
	**5 signals among 20 variables**		
One-at-a-time*	0.09 (0.01)	0.58 (0.02)	0.40 (0.02)
Stepwise	<0.01 (<0.01)	<0.01 (<0.01)	0.01 (<0.01)
Two-step BVS*	<0.01 (<0.01)	<0.01 (<0.01)	<0.01 (<0.01)
	**5 signals among 100 variables**		
One-at-a-time*	0.25 (0.02)	0.77 (0.02)	0.67 (0.02)
Stepwise	0.01 (<0.01)	0.03 (0.01)	0.08 (0.01)
Two-step BVS*	<0.01 (<0.01)	0.01 (<0.01)	0.01 (<0.01)
	**5 signals among 1000 variables**		
One-at-a-time*	0.53 (0.03)	0.93 (0.01)	0.78 (0.02)
Stepwise	0.07 (0.01)	0.14 (0.01)	0.27 (0.01)
Two-step BVS*	0.01 (<0.01)	0.01 (<0.01)	0.06 (0.01)

Mean false discovery rate, the proportion of noise variables selected, is displayed for 200 simulations with the corresponding Monte Carlo errors in brackets.

*Selection thresholds chosen to match the sensitivity of the stepwise method in each simulation, for which a nominal *P*-value inclusion threshold of 0.05 was used.

Relative patterns of performance among the frameworks were not materially changed when a more liberal *P*-value inclusion threshold of 0.1 was used in the stepwise selection procedure (Supplementary Tables 1 and 2, available as Supplementary data at *IJE* online); the BVS method continued to offer the best discrimination of signal to noise variables in terms of both sensitivity and specificity.

## Application of the methods to data from the EPIC-InterAct case-cohort study

We exemplify the three variable selection methods using data from 777 incident type 2 diabetes (T2D) cases and a subcohort of 972 individuals (including 28 of the T2D cases) from one of the centres (Cambridge, sampled from 23 081 individuals with stored blood) contributing to the EPIC-InterAct case-cohort study. In this example, each individual has values of nine saturated fatty acids and 11 polyunsaturated fatty acids, measured in plasma phospholipids. The prospective associations between each of these fatty acids and incident T2D have already been published for the full EPIC-InterAct study;^26^^,^^27^ further information about the study design is also included in these papers.

**Figure 1:**
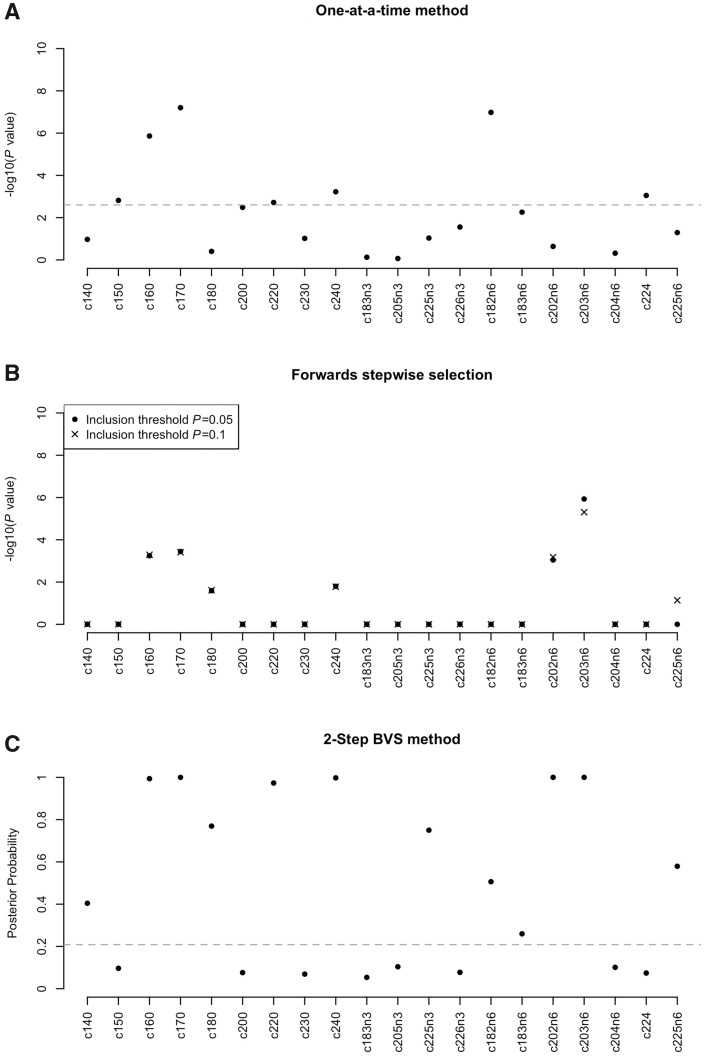
Results from application of three variable selection methods to data from the EPIC-InterAct case-cohort study. Panel A) shows the log10(P value) for each fatty acid from one-at-a-time Prentice-weighted Cox regression models; the dashed line indicates the Bonferroni significance threshold (0.05/20 =0.0025). Panel B) shows the log10(P values) for the combination of fatty acids selected using the stepwise method according to inclusion thresholds of P=0.05 and P=0.1. Panel C) shows posterior probabilities of selection using the BVS method; the dashed line indicates a Bayes Factor of 5.

Figure 1 presents the –log10(*P-*value) for each fatty acid from the one-at-a-time and stepwise methods, and posterior probabilities of selection from the BVS method. All analyses were adjusted for age and sex. Eight fatty acids were significant according to a Bonferroni threshold (0.05/20 = 0.0025) using the one-at-a-time method, whereas the forwards stepwise algorithm selected six fatty acids using a *P*-value inclusion threshold of 0.05 and an additional seventh fatty acid using an inclusion threshold of 0.1. Accounting for correlations in the multivariable stepwise framework, four fatty acids were ruled out under both inclusion thresholds, and two fatty acids were included which had not been significant using the one-at-a-time method: c202n6 and c180. There were substantial correlations between the fatty acids (Supplementary Figure 1, available as Supplementary data at *IJE* online) which, in the presence of multiple signals, can cause both exaggeration and attenuation of signals in one-at-a-time analysis.

The significant associations revealed for c202n6 and c180 when accounting for correlations were further supported by the BVS method, which also provided strong evidence of association for both these fatty acids. Notably, c202n6 had a *P*-value of 0.23 from the one-at-a-time method [HR: 0.94 95% confidence interval (CI): (0.85, 1.04)] but was significant using the BVS method [HR: 0.80 95% CI: (0.69, 0.92), posterior probability 1, Bayes factor ∞]. In total, 12 fatty acids had significant inclusion probabilities (based on Bayes Factor > = 5) using the BVS method. There was strong evidence for two additional fatty acids which were not significant using the one-at-a-time or stepwise methods: c140 and c225n3. Table 4 shows the estimated associations for the 12 fatty acids selected by the BVS method.

**Table 4.** Hazard ratios, 95% CIs, posterior probabilities and Bayes Factors for the fatty acids selected using the BVS method

Fatty acid	HR (95% CI)^a^	Posterior probabilities	Bayes Factor
Saturated:			
c160	1.25 (1.04, 1.50)	1.00	7672.3
c170	0.77 (0.66, 0.89)	1.00	∞
c180	1.17 (1.00, 1.38)	0.75	60.6
c220	1.25 (0.91, 1.71)	1.00	6646.7
c240	0.67 (0.50, 0.90)	1.00	∞
n-3 polyunsaturated:			
c225n3	0.88 (0.75, 1.03)	0.74	55.6
n-6 polyunsaturated:			
c182n6	0.85 (0.70, 1.02)	0.60	30.2
c202n6	0.79 (0.68, 0.91)	1.00	∞
c203n6	1.44 (1.22, 1.70)	1.00	∞

Hazard ratios are per 1 standard deviation in the fatty acid.

^a^Confidence intervals for 3/9 fatty acids in the modal model included 1. The fatty acids were selected for inclusion using the BVS algorithm based on logistic regression, and hazard ratios were estimated from Prentice-weighted Cox regression. As shown in the simulations, the BVS algorithm has higher sensitivity than methods using weighted Cox regression, so it is possible that the CIs around the hazard ratios for some variables selected using BVS will include 1.

## Discussion

We have described a two-step method for variable selection in case-cohort studies, combining a multivariable BVS algorithm in the first step with Prentice-weighted Cox regression in the second step. In simulations based on artificial datasets including up to 1000 variables, this method provided substantial improvements in sensitivity and false discovery rate compared with either one-at-a-time or simple stepwise approaches. We have also demonstrated the applicability of the method to real data from a case-cohort study.

The BVS method uses a logistic regression model in the first step, which ignores time to event data. Our simulations and real data example both indicate that any power loss from ignoring time to event data is outweighed by the use of a superior variable selection algorithm, relative to stepwise selection using multivariable weighted Cox models. This is consistent with Staley *et al.* (submitted), who report similar findings comparing logistic and Prentice-weighted Cox regression analyses of data from genome-wide association studies, and other recent work that suggests the efficiency loss from using logistic regression compared with Cox regression is low.^28^

A penalized regression-based variable search strategy was recently described for a likelihood function modified for a case-cohort study.^29^ However, without associated published software, it seems unlikely that this method will be a viable option for applied researchers and so we did not consider it further here. Moreover, an attractive feature of BVS compared with Lasso-type penalized regression methods, and why we explored BVS here, is that interpretable measures of significance are obtained for all variables. This would enable prioritization of significant findings for planning follow-up studies.

The variable selection frameworks we explored assumed additive models, i.e. searched for main effects of continuous or binary variables but not interactions. Searching for interactions in a high-dimensional variable space is a very challenging problem. In the future we plan to explore extensions to the BVS framework incorporating recently proposed sparse regression methodology capable of exploring interactions, such as that by Lim and Hastie.^30^ Searching over variables with multiple categories would also require a modification of the current algorithm. However, any number of known confounders (including categorical variables) may be accounted for by fixing them in the BVS model while searching over other predictors (which is equivalent to ascribing the confounders a prior inclusion probability of one).

In summary, we propose that the two-step BVS method should be used in analysis of a case-cohort study where the aim is to detect associations of multiple variables with the outcome. Software to implement the method is freely available in the R2BGLiMS R package, available via github, and an example script is provided in the Appendix, available as Supplementary data at *IJE* online.

## Supplementary Data

Supplementary data are available at *IJE* online.

## Funding

P.J.N. and S.R. were supported by the Medical Research Council [www.mrc.ac.uk] (Unit Programme number MC_UP_0801/1). P.J.N. also acknowledges partial support from the NIHR Cambridge Biomedical Research Centre. S.S. and S.C. were supported by the Medical Research Council [www.mrc.ac.uk] (Unit Programme number MC_U105260558). S.J.S. was supported by the Medical Research Council [www.mrc.ac.uk] (Unit Programme number MC_UU_12015/1). Funding for the EPIC-InterAct project was provided by the EU FP6 programme (grant number LSHM_CT_2006_037197).

## Supplementary Material

Supplementary Data

Supplementary Appendix
