# Supplementary material for: A two-step method for variable selection in the analysis of a case-cohort study
Source: Int J Epidemiol. 2017 Nov 10;47(2):597–604. doi: 10.1093/ije/dyx224 (PMC5913627; doi:10.1093/ije/dyx224)
Supplement: Supplementary Data [file dyx224_ije-2017-02-0160-file004.pdf]

Supplementary Table 1. Sensitivity of variable selection methods, for each scenario, at the false discovery rate of stepwise selection using a 0.1 *P* value inclusion threshold.

**Method**	**Pairwise correlation between all variables**		
	**0.2**	**0.5**	**0.8**
		**5 signals among 20 variables**	
One-at-a-time*	0.77 (0.01)	0.48 (0.01)	0.45 (0.01)
Stepwise	0.80 (0.01)	0.77 (0.01)	0.67 (0.01)
2-Step BVS*	0.89 (0.01)	0.85 (0.01)	0.75 (0.01)
		**5 signals among 100 variables**	
One-at-a-time*	0.71 (0.01)	0.44 (0.01)	0.41 (0.01)
Stepwise	0.81 (0.01)	0.76 (0.01)	0.64 (0.01)
2-Step BVS*	0.84 (0.01)	0.79 (0.01)	0.68 (0.01)
		**5 signals among 1000 variables**	
One-at-a-time*	0.65 (0.01)	0.42 (0.00)	0.39 (0.01)
Stepwise	0.79 (0.01)	0.74 (0.01)	0.63 (0.01)
2-Step BVS*	0.81 (0.01)	0.76 (0.01)	0.66 (0.01)

Mean sensitivity, the proportion of true signals selected, is displayed for 200 simulations with the corresponding Monte Carlo errors in brackets. *Selection thresholds chosen to match the false discovery rate of the stepwise method in each simulation, for which a nominal *P* value inclusion threshold of 0.1 was used.

Supplementary Table 2. False discovery rates of variable selection methods, for each scenario, at the sensitivity of stepwise selection using a 0.1 *P* value inclusion threshold.

**Method**	**Pairwise correlation between all variables**		
	**0.2**	**0.5**	**0.8**
		**5 signals among 20 variables**	
One-at-a-time*	0.14 (0.01)	0.68 (0.02)	0.54 (0.02)
Stepwise	0.01 (<0.01)	0.02 (<0.01)	0.04 (0.01)
2-Step BVS*	<0.01 (<0.01)	<0.01 (<0.01)	<0.01 (<0.01)
		**5 signals among 100 variables**	
One-at-a-time*	0.34 (0.02)	0.86 (0.02)	0.77 (0.02)
Stepwise	0.05 (0.01)	0.10 (0.01)	0.21 (0.01)
2-Step BVS*	<0.01 (<0.01)	0.01 (<0.01)	0.03 (0.01)
		**5 signals among 1,000 variables**	
One-at-a-time*	0.61 (0.03)	0.96 (0.01)	0.91 (0.01)
Stepwise	0.29 (0.01)	0.44 (0.01)	0.64 (0.01)
2-Step BVS*	0.02 (0.01)	0.05 (0.01)	0.19 (0.02)

Mean false discovery rate, the proportion of noise variables selected, is displayed for 200 simulations with the corresponding Monte Carlo errors in brackets. *Selection thresholds chosen to match the sensitivity of the stepwise method in each simulation, for which a nominal *P* value inclusion threshold of 0.1 was used.

Supplementary Figure 1. Correlations between fatty acids in the EPIC-InterAct case-cohort study. Correlations are calculated using data from the subcohort.

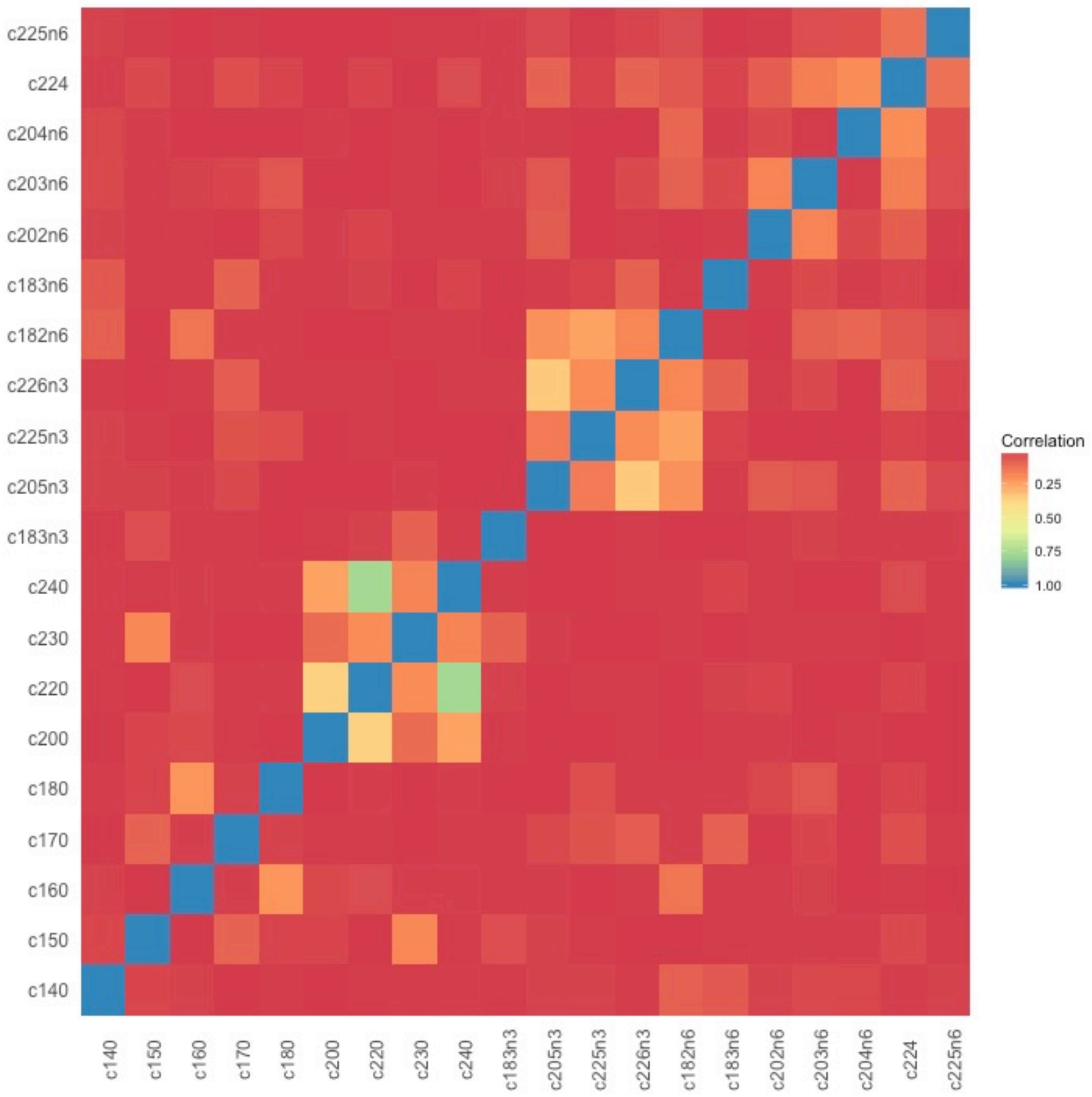

Pairwise Pearson r^2 are displayed.
