# Supplementary material for: A two-step method for variable selection in the analysis of a case-cohort study
Source: Int J Epidemiol. 2017 Nov 10;47(2):597–604. doi: 10.1093/ije/dyx224 (PMC5913627; doi:10.1093/ije/dyx224)
Supplement: Supplementary Appendix [file dyx224_appendix.pdf]

Appendix: Example of running the 2-step BVS method in R

```
############################
# --- Install R2BGLiMS --- #
############################

# The underlying algorithm is coded in Java. Therefore you must have the Java JDK #
#(v8 or later) installed: https://www.java.com/download/
# After installing the java JDK, intall R2BGLiMS using the “devtools” package:
install.packages("devtools")
library(devtools)
install_github("pjnewcombe/R2BGLiMS")

################################
# --- Step 1: Logistic BVS --- #
################################

library(R2BGLiMS)
data("CaseCohortExample") # Only V1,V2,V3,V4,V5 have true effects.
# Run Bayesian logistic analysis for 0.2 million iterations
n.mil.its <- 0.2
for (v in covariate.names) { data.cc[,v] <- data.cc[,v] - mean(data.cc[,v]) }
logistic.cc.res <- R2BGLiMS(
  likelihood="Logistic",
  data=data.cc,
  outcome.var="event",
  model.space.priors=list("a"=1,"b"=length(covariate.names),
     "Variables"=covariate.names),
  n.mil=n.mil.its
)
# Diagnostic trace plot of log-likelihood
plot(logistic.cc.res@mcmc.output[,"LogLikelihood"], type="l") # Looks ok
# Summary plot of selecion probabilities
ManhattanPlot(logistic.cc.res)
# Results table
logistic.cc.res@posterior.summary.table

##############################################################
# --- Step 2: Effect estimation using the Prentice model --- #
##############################################################

library(survival)
# Fit a Prentice weighted Cox regression model using covariates with BF>5
top.vars <-
names(which(logistic.cc.res@posterior.summary.table[covariate.names,"BF"]>5))
prentice.model.formula <- as.formula(paste("Surv(times, event) ~ ",paste(top.vars,
collapse="+")))
prentice.res <- cch(
  prentice.model.formula,
  data = data.cc,
  subcoh = ~subcohort,
  id=~ID,
  cohort.size=n.complete.cohort,
  method="Prentice")
summary(prentice.res)
```
